# Copper as an antimicrobial agent: recent advances

**DOI:** 10.1039/d1ra02149d

**Published:** 2021-05-19

**Authors:** Intisar Salah, Ivan P. Parkin, Elaine Allan

**Affiliations:** Materials Chemistry Research Centre, Department of Chemistry, University College London 20 Gordon Street London UK; Department of Microbial Diseases, Eastman Dental Institute, University College London Royal Free Campus, Rowland Hill Street London UK e.allan@ucl.ac.uk

## Abstract

From its uses in ancient civilisations, copper has an established history as an antimicrobial agent. Extensive research has determined the efficacy and mechanism of copper's antimicrobial activity against microorganisms. The process is multifaceted with the main mechanism of bactericidal activity being the generation of reactive oxygen species (ROS), which irreversibly damages membranes. Copper ions released from surfaces lead to RNA degradation and membrane disruption of enveloped viruses. For fungi, the mechanism involves the physical deterioration of the membrane and copper ion influx. Due to variations in the experimental parameters, it is difficult to compare studies directly. In this review article, we outline the importance of the experimental conditions currently employed and how they bear little resemblance to real-world conditions. We endorse previous recommendations calling for an update to industrial standard tests.

## Abstract 2

The causes of death for each microorganism by CuNPs and metallic copper is summarised.

## Graphical abstract

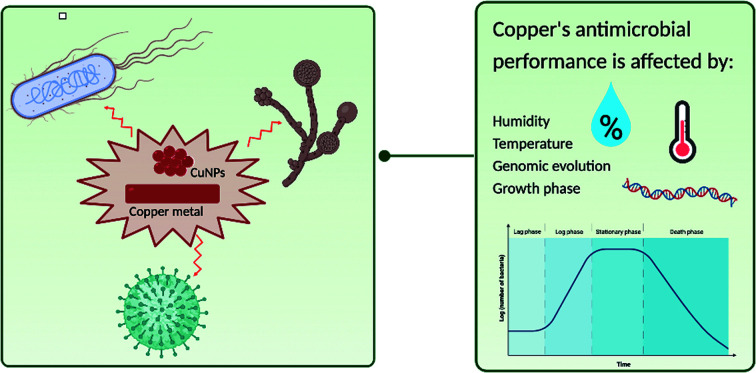

## Introduction

The ability of transition metals and metalloids to non-specifically target bacteria, viruses and fungi make them attractive antimicrobials. Specifically, copper is an esteemed antimicrobial agent and was utilised in ancient civilisation for its properties in medicinal products and water vessels before an understanding of the role of microbes in biofouling were acquired.^1^ Improvements in nanotechnology in recent years has led to a focus on developing copper nanoparticles (CuNPs) for antimicrobial performance. They have a substantially larger surface-area-to-volume ratio resulting in increased toxicity compared to the metal, alongside improved optical properties making them a desirable replacement.^2–5^

Pathogenic microbes may be transmitted directly from person-to-person or indirectly *via* contamination of surfaces leading to both healthcare-acquired infections (HAIs) and community-acquired infections (CAIs).^6–8^ The exact mechanism of microbial death from copper is controversial and the significance and relative contribution of each proposed mechanism are unclear. One method is the physical interaction of the CuNPs with the cell, or virus, plasma membrane leading to its destruction, making the microbe susceptible to damage from copper ions.^9–15^ The smaller nanoparticles (NPs), between 1–10 nm, favour this mechanism as they can attach to the membrane and infiltrate the cell.^16,17^ Another method of copper's action is the generation of reactive oxygen species (ROS) by reduction of copper through a Fenton-like reaction, leading to enzyme and non-enzyme mediated oxidative damage involving lipid peroxidation, protein oxidation and DNA damage.^18–20^ The final mechanism is the release of copper ions, Cu^+^ and Cu^2+^, which damage the membrane and infiltrate the cell, inducing an oxidative stress response involving endogenous ROS.^21–23^ The consensus view of the cause of microbial cell death due to copper is a combination of these processes with the relative importance of each dependent on the microorganism; this will be explored in this paper.

The relative contributions of copper ions and ROS to efficacy can be ascertained by performing experiments in the presence and absence of chelating agents and quenching agents, respectively. Ethylenediaminetetraacetic acid (EDTA) is a chelating agent that forms complexes with Cu^2+^ released from the metal, and bathocuproine disulfonic acid complexes with Cu^+^. A commonly used agent to quench hydroxyl radicals is d-mannitol and for superoxide anions, 4,5-dihydroxy-1,3-benzene disulfonic acid. DNA integrity can be assessed by gel electrophoresis to separate DNA fragments. Membrane integrity can be visualised using a fluorescent live/dead stain which displays the viable and non-viable cells as green and red, respectively, using a fluorescence microscope.^24,25^ However, since permeability can be transient, fluorescent dyes alone cannot determine the extent of the damage and a quantitative assessment of the ability of the microorganism (bacteria or yeast) to form colonies is often employed using stainless steel as a negative control.^26–29^

## Activity against bacteria

**Fig. 1:**
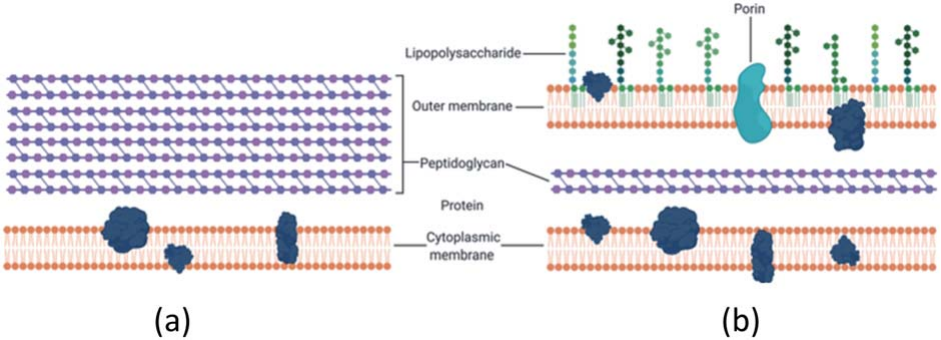
(a) Gram-positive bacteria possess a characteristic thick peptidoglycan layer. (b) Gram-negative bacteria exhibit an outer membrane and a thinner peptidoglycan layer.

There is a distinction between the Gram-negative and Gram-positive bacterial response to copper potentially due to structural differences as seen in Fig. 1. Gram-negative bacteria have an outer membrane which makes them less susceptible to antibacterial agents.^17,30,31^ The normal mode of bacterial growth is in the form of a biofilm where the bacterial cells are adherent to a surface (or each other) and surrounded by a self-produced exopolymeric matrix comprising of polysaccharides, proteins and nucleic acids. Bacteria growing in a biofilm are challenging to remove, display increased resistance to antimicrobial agents compared to planktonic bacteria and are associated with medical device-related and tissue-related infections including urinary tract infections (UTIs), pneumonia and chronic wound infections.^32–36^

### Activity against *Escherichia coli*

Pathogenic *Escherichia coli* is a common cause of HAIs and CAIs, including UTIs, and gastrointestinal disease, amongst other illnesses.^37,38^*E. coli* is often used as the model Gram-negative bacterium to test bactericidal efficacy. *E. coli* is sub-divided into pathotypes which differ in their complement of accessory genes (*i.e.*, virulence factors) but share core genomes. Different pathotypes are associated with different diseases. For instance, O157:H7 is a foodborne pathogen and responsible for diarrhoea and haemorrhagic colitis, as well as the life-threatening haemolytic-uremic syndrome.^39^*E. coli* K12 is a domesticated strain known for being one of the first isolated microbes but is also notorious for being ‘far from [the] model’ microbe found in the clinical setting.^40^ This is because it is non-pathogenic and is therefore commonly employed in research as a model bacterium.

### Copper surface

The behaviour of three strains of *E. coli*, a pathogenic O157:H7 strain, and two K12 strains, on a copper surface were studied.^41^ Using a dry inoculum, the pathogenic strain survived for longer than the other two strains but all were non-viable after 10–20 minutes of exposure to the copper surface in comparison to stainless steel which still had live bacteria after 30 minutes. Staining with rhodamine 123 (where cells with intact membranes fluoresce) showed that fluorescence was rapidly lost on exposure to the copper surface indicating membrane disruption. Protection was detected by copper ion chelators alongside d-mannitol, which offered more protection than the superoxide anion quencher, suggesting most of the ROS present were hydroxyl radicals. This confirms the generation of ROS by a mechanism independent of the Fenton reaction, as proposed by the authors.

### Copper nanoparticles

In a study where *E. coli* ATCC 15224 was exposed to increasing concentrations of 12 nm CuNPs, the authors reported a progressive inhibition of bacterial growth with increasing concentration.^42^ Cu^2+^ ion release was measured and increased proportionally with the increased concentration of CuNPs suggesting that ion release is important for activity. The small size of the nanoparticles makes them easy to penetrate the cells and, through scanning electron microscopy (SEM), the authors demonstrated a shift in the morphology of the rod-shaped *E. coli* to irregular shapes in the presence of copper. This suggests that the CuNPs interact with the cell wall and damage the plasma membrane affecting its integrity as proposed by previous authors.^43–45^ However, the role of ROS was not investigated.

In another study, evidence was presented for the role of Cu^2+^ ions in the degradation of *E. coli* as the size of CuNPs were reduced from 62.5 nm to 23.4 nm in the presence of EDTA for 30 minutes.^46^ Presumably, this was a result of gradual leaching (and subsequent chelation) of copper ions from the NP surface as the CuNPs left in suspension for 1 hour revealed no decrease in size. The generation of ROS inside bacterial cells were studied using the 2′,7′-dichlorodihydrofluorescein diacetate (DCFH-DA), a reduced form of fluorescein that is oxidised and highly fluorescent in the presence of ROS. *E. coli* K12 was exposed to CuNPs sized 62.5 nm for 1 hour at the minimum inhibitory concentration and the minimum bactericidal concentration which resulted in 1.8 and 2.5 times, respectively, overproduction of cellular reactive singlet oxygen and hydroxyl radicals in comparison with untreated cells. This study used *E. coli* K12 and studies with different *E. coli* strains have confirmed that even at low concentrations, CuNPs show good activity by invoking a strong ROS response.^46–48^

### Activity against *Staphylococcus aureus*

*Staphylococcus aureus* is a major cause of skin and soft tissue infections.^49,50^ After the introduction of meticillin, resistant strains rapidly evolved^51^ and meticillin-resistant *S. aureus* (MRSA) has become a major problem in hospitals and increasingly in the community. Hence, research has focused on finding ways to reduce its transmission between patients.^52^

### Copper surface

In a study exposing MRSA to copper metal surfaces, complete kill was observed after 20 μL drops of bacterial suspension (containing 10^7^ CFU) were exposed for 90 minutes at 22 °C in air, for three different strains including representatives of the epidemic clones, EMRSA-1 (NCTC 11939) and 16 (NCTC 13143).^27^ In comparison, stainless steel had viable cells after 72 hours of exposure, as anticipated. A notable finding was that there was no significant difference in the numbers of surviving bacteria between samples recovered with or without a Cu^2+^ chelator. This suggests that another mechanism independent of the presence of Cu^2+^ ions is responsible for the bactericidal activity of the copper surfaces.

**Table 1:** Antimicrobial activity of copper suspensions and copper-containing surfaces against microorganisms^a^

Test organism & strain	Inoculum	Antimicrobial agent & UNS name	NP concentration & size	Inactivation time	Primary/significant MoA	Ref.
*E. coli* O157:H7	1 × 10^7^ CFU	Cu surface (C11000)	—	10 min	ROS not *via* Fenton	41
*E. coli* K12	1 × 10^7^ CFU	Cu surface (C11000)	—	10 min		
*E. coli*-ATCC 15224	<1.0 × 10^8^ CFU	CuNP suspension	20–100 μg mL^−1^, 12 nm	Not specified	Physical interaction	42
*E. coli* K12	1 × 10^8^ CFU	CuNP suspension	3.0 & 7.5 μg mL^−1^ 62.5 nm	1 h	ROS *via* Fenton	46
MRSA-NCTC 10442	1 × 10^7^ CFU	Cu surface (C19700)	—	45 min	ROS not *via* Fenton	27
EMRSA-1 NCTC 11939	1.9 × 10^7^ CFU	Cu surface (C19700)	—	60 min		
EMRSA-16 NCTC 13143	1.5 × 10^7^ CFU	Cu surface (C19700)	—	90 min		
EMRSA-16 NCTC 13143	1 × 10^7^ CFU	Cu surface	—	20 min		53
MSSA-ATCC 49230	1 × 10^7^ CFU	Cu surface	—	15 min		
*S. aureus*-ATCC 6538	1 × 10^5^ CFU	CuNP suspension	200–3200 μg mL^−1^, 9 nm	16 h	Physical interaction	54
SARS-CoV-2 – USA-WA1/2020	5 μm × 10^5.25^ TCID_50_/mL	Cu surface	—	4 h	RNA degradation	29
HuCoV-229E	1 × 10^3^ PFU	Cu surface (C11000)	—	60 min		^62^
Influenza A H1N1	2 × 10^6^ PFU	Cu surface (C11000)	—	6 h	Cu ions cause RNA degradation	28
Norovirus-murine norovirus	5 × 10^4^ PFU	Cu surface (C11000)	—	30 min	Cu ions, particularly, Cu^+^, cause RNA degradation	65
*Aspergillus* spp.	1 × 10^6^ spores	CuNP suspension	100 μg mL^−1^, 25.5 nm	5 days	Physical interaction, copper ions	71
*C. albicans* SC5314, CAF3-1, CaCUP1, CaCRP1	12.5 × 10^6^ CFU	Cu surface (C11000)	—	60 min (mutants 5–10 min)		9
*Candida* spp., *C. Albicans* ATCC MYA-2876, *C. glabrata* ATCC 2001, *C. tropicalis* ATCC 750	1–5 × 10^5^ CFU	CuONP suspension	25–200 mM, 35 nm	Not specified		73

a UNS: unified numbering system for metals and alloys; MoA: mechanism of action; ref: reference.

Another study applied smaller droplets (1 μL containing 10^7^ CFU) of the same EMRSA-16 strain (NCTC 13143) to examine the activity of a copper surface compared to a meticillin-sensitive *S. aureus* (MSSA).^53^ The authors reported complete kill of EMRSA in 20 minutes and MSSA in 15 minutes. The addition of a chelating agent and a superoxide anion quencher protected the bacteria; however, the addition of a hydroxyl quencher and a hydrogen peroxide-decomposing agent offered no protection. This indicated there was ROS generation, however not *via* Fenton chemistry.

### Copper nanoparticles

Betancourt-Galindo and co-workers assessed the effect of exposure to increasing concentrations of 9 nm CuNPs on *S. aureus* ATCC 6538.^54^ As expected, increasing CuNP concentration resulted in a progressive increase in growth inhibition and the highest test concentration (3200 μg mL^−1^) was required for complete inhibition of growth. In contrast, 1600 μg mL^−1^ was sufficient for complete inhibition of the Gram-negative bacterium, *Pseudomonas aeruginosa*. Although there was no attempt to elucidate the mechanism, we can speculate that the presence of a thicker peptidoglycan layer in *S. aureus* compared to *P. aeruginosa* may be responsible for reduced cellular penetration by the CuNPs.

## Activity against viruses

Viruses have either DNA or RNA genomes and the nucleic acid is either double-stranded or single-stranded, respectively. RNA viruses are more likely to cause outbreaks of infections as they have a higher mutation and evolutionary rates making them more virulent.^55^ In this article, we review the literature on the effect of copper surfaces on the RNA viruses, SARS-CoV-2, influenza A and norovirus. Severe acute respiratory syndrome coronavirus 2 (SARS-CoV-2) is the highly infectious virus responsible for the COVID-19 pandemic. It is spread through droplets exhaled from infected people and through aerosols propelled by coughs and sneezes.^56,57^ Influenza A causes the common flu and can lead to pneumonia and high fever.^58^ It has a high morbidity and mortality rate amongst high-risk groups, particularly the elderly. Norovirus, also known as the ‘winter vomiting bug’ is also common, responsible for epidemic gastroenteritis and annually costs the NHS £100 million.^59,60^ Therefore, solutions to reduce the spread of this highly contagious virus is in high demand. Because of the risk of handling highly infectious viruses like SARS-CoV-2, less infectious surrogates are often used and potential biological differences between the actual pathogen and its surrogates should be borne in mind when the results are interpreted.

### Activity against SARS-CoV-2

Recent studies have demonstrated that SARS-Cov-2 is stable on copper surfaces for up to 4 hours after exposure.^29,61^ This is in comparison to stainless steel and plastic surfaces where the virus survives for up to 48 and 72 h, respectively.^29^ The strain used in this study serves as the reference for SARS-CoV-2.^62^ Another study demonstrated the degradation of genomic RNA of another human coronavirus (HuCoV-229E) by copper.^63^ These authors also examined copper ion release by using copper ion chelators which protected the virus for up to 2 hours suggesting both ions are involved in the virucidal mechanism. They also assessed the generation of ROS by using d-mannitol and a superoxide anion quencher. They found that the latter protected the virus for a considerably longer period of time compared to exposure in the presence of d-mannitol, suggesting superoxide radicals are significant in the inactivation of this coronavirus. As a negative control, the authors used the same chelating and quenching agents on stainless steel and reported no significant effect.

### Activity against influenza A

Research comparing the rate of inactivation of influenza A on a copper surface with stainless steel showed promising results.^28^ This study demonstrated a nearly 4-log reduction of infectious particles on the copper surface after just 6 hours of incubation at room temperature compared to a 1-log reduction after 24 hours on stainless steel. The authors suggest the degradation of the genomic material may be responsible for the activity although no supporting evidence was presented.

### Activity against norovirus

The first study to look at the antiviral properties of a copper surface against a surrogate murine norovirus reported zero-counts of virus particles after only 30 minutes of exposure.^64^ The murine surrogate is phylogenetically closest to the human norovirus and is regarded as an appropriate model.^65^ Analysis of genomic RNA by gel electrophoresis showed complete genome degradation following exposure to copper. To study the mechanism, quenchers and chelators were utilised. The results demonstrated that the quenchers did not affect virus inactivation whereas the chelators offered protection. Due to the lack of action of the quenching agents, it can be inferred that ROS generation was not the primary mechanism and instead copper ions contributed significantly to virucidal activity.

## Activity against fungi and yeast

Fungi are a diverse group of microorganisms including both yeast and filamentous fungi and are a cause of subcutaneous, cutaneous and systemic disease and if not treated, can result in chronic infections.^66^*Aspergillus* is a filamentous fungus that produces spores which are ubiquitous in the environment. Inhalation of spores can lead to fatal systemic infections in immunocompromised hospital patients.^67,68^ Several studies have concentrated on *Candida* sp., the genus of yeast responsible for candidiasis, a common fungal infection.^67,69^ Nearly a million cases of invasive and potentially deadly candidiasis are diagnosed annually and cases are on the rise, particularly in immunocompromised individuals, including patients undergoing cancer chemotherapy, transplants and haemodialysis.^67,70^*C. albicans*, *C. glabrata*, and *C. tropicalis* are the three species collectively responsible for 83.8% of candidiasis cases.^71^

### Activity against *Aspergillus* species

In one study, numerous species, including opportunistic *A. flavus* and *A. terreus*, were presented with 25.5 nm CuNPs and incubated for 5 days at 28 °C in air.^71^ Relatively long periods of exposure are required because of the slow growth rate of the fungi.^72^ The parameters set in this experiment were sufficient to achieve 58–73% inhibition of fungal growth although whether this reflects inhibition of spore germination or growth of vegetative filaments is not clear. Agarose gel electrophoresis of genomic DNA isolated after exposure to NPs indicated fragmentation of the genome suggesting NP penetration of the cell wall.

### Activity against *Candida* species

One study exposed *C. albicans* to a copper surface and found the yeast was inactivated in 5 minutes at 23 °C.^9^ A fluorescent dye, dihydroethidium, was employed to detect cytoplasmic ROS within 1 minute of exposure alongside a copper-sensitive dye that exhibited an uptake of copper ions. A mutant lacking the copper ion efflux system showed increased susceptibility to copper compared to the wild-type strain, demonstrating the importance of copper ions in the fungicidal activity. Live/dead staining showed the membranes were damaged instantly on exposure to the copper surface, suggesting the physical interaction of CuNPs with the yeast cell is vital in the mechanism of yeast cell death.

Another study prepared 35 nm CuONPs and demonstrated a 53% inhibition of growth for *C. albicans* and *C. glabrata*, and 59% for *C. tropicalis*.^73^ An alteration of the cellular morphology was evident by SEM. Although this is a promising demonstration of the potential of CuONPs in preventing yeast infections, further studies are required to elucidate the precise mechanism of growth inhibition.

## Summary of copper's activity against microorganisms

**Fig. 2:**
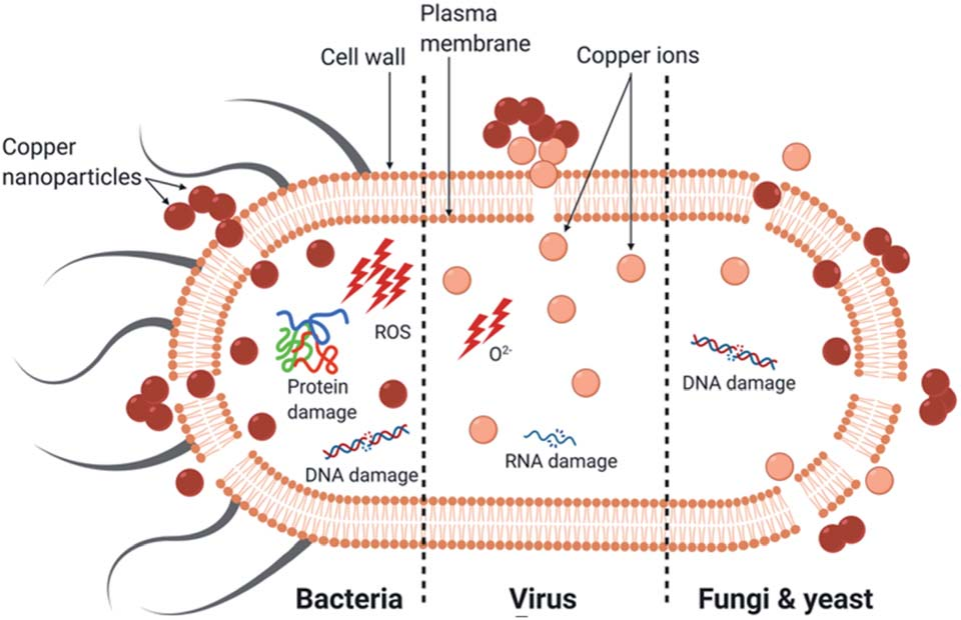
The primary mechanism of death in different microorganisms by copper nanoparticles.

In summary, copper has an intrinsic antimicrobial effect on bacteria, viruses and fungi. The activity of CuNP appears to depend more on their size rather than their concentration: the smaller the nanoparticles, the greater the efficacy.^46–48^ The primary causes of death for each microorganism is presented in Fig. 2. The main mechanism of bactericidal activity is the generation of ROS, both dependent and independent from Fenton chemistry, and results in membrane damage. The principal mechanism of activity of copper surface against viruses was ion release leading to RNA degradation and membrane disruption in the case of enveloped viruses. For fungi, the uptake of copper ions and the physical deterioration of the membrane leading to copper influx are considered to be the primary mechanisms (Table 1).

## Antimicrobial properties of copper alloys

Commonly used copper alloys are brass and bronze, the former consisting of copper and zinc and the latter, copper and other metals, typically tin, aluminium, nickel and metalloids such as silicon.^26^ The copper alloys also have antimicrobial activity and studies have shown that there is a direct correlation between the copper content and antimicrobial efficacy.^26,74–76^ Alongside improving the antimicrobial activity, changing the composition of metals can affect other properties; for instance, the ratio of copper-to-zinc in brass can be altered to ensure the surface is sufficiently hard for applications such as hospital furniture and doorknobs.^77^

### Brass

Due to zinc's inherent antimicrobial activity, when combined with copper, there is a synergy between the two metals, therefore, even at low copper concentrations, there is notable antiviral activity.^63,78^ Nickel, on the other hand, has no intrinsic antimicrobial properties, thus, the increase in copper content has a proportional impact on activity. A comparison of brass and CuNi, both with 70% copper, revealed that brass inactivates human coronavirus (HuCoV-229E) faster.^63^ After 30 minutes, the authors reported a 4-log reduction in virus particles compared to only 1-log reduction after 2 hours on CuNi. In another study, brass with 86% and 63% copper also demonstrated bactericidal activity against *E. coli* at higher copper concentrations; it took 15 minutes for the 86% copper and 30 minutes for 63% copper to achieve full inactivation of bacteria.^26^ The synergy between copper and zinc has demonstrated that brass can be a cost-effective option for large surfaces such as those required in hospitals.^26^ Another study looked at the antibacterial performance of brass (80% copper) against MRSA and reported a 4-log reduction in bacterial numbers within 3 hours compared to no significant reduction on stainless steel after 6 hours.^27^

In a 10 week study in a busy British hospital, plastic, chrome-plated and aluminium surfaces were replaced with brass, of 60% or 70% copper.^79^ In comparison to traditional surfaces, brass resulted in 90–100% inactivation of MSSA, vancomycin-resistant enterococcus (VRE) and *E. coli*. Another study similarly replaced hospital surfaces with copper alloys and showed that the reduced surface colonisation by bacteria and yeast was associated with a 58% decrease in the incidence of HAIs.^80^ Although the exact alloys were not disclosed in this study, the results are promising and demonstrate the potential of copper alloys potential as antimicrobial agents for surface disinfection in hospitals.

### Bronze

Three bronze surfaces, C65500 (97% copper, 3% silicon), C61500 (90% copper, 8% aluminium, 2% nickel) and C51000 (95% copper, 5% tin) were exposed to *E. coli* O157:H7 (ref. 81) and a reduction in bacterial numbers to below the detection limit in 65, 180 and 105 minutes, respectively, was reported. Silicon has proven to have antibacterial and antifungal properties and tin is a powerful antimicrobial agent which means when these elements are combined with copper to form bronze, they reduce the inactivation time as seen in C65500 and C51000.^82–85^ Although aluminium has intrinsic antimicrobial properties, C61500 took the longest to reduce bacterial numbers to below the detection limit suggesting that a higher copper content is required for maximum activity.^86,87^

## Summary of copper alloys' activity against microorganisms

Brass and bronze present excellent antimicrobial efficacy and the activity of the alloys increases proportionally with the concentration of copper. The suggested antimicrobial mechanism is copper ion release supporting the Fenton reaction leading to the production of hydroxyl radicals.^63^ This was established by using copper ion chelators, with brass and copper exposed to a human coronavirus (HuCoV-229E). The chelators protected the virus by increasing the time taken for inactivation, indicating that the release of copper ions is important in the inactivation mechanism. There is also evidence to determine DNA degradation is not necessarily a cause of bacterial cell death.^12^ The relative contributions of membrane damage, penetration into the cytoplasm of bacteria and fungi, and the generation of ROS by copper alloys need to be elucidated (Table 2).

**Table 2:** Antimicrobial activity of copper alloys against microorganisms^a^

Test organism & strain	Inoculum	Antimicrobial surface & UNS name	% Copper	Extent of reduction	Inactivation time	Ref.
Murine norovirus-HuCoV-229E	5 × 10^5^ PFU	CuZn (C26000)	70	4-log	30 min	62
		CuNi (C71500)	70	1-log	2 h	
*E. coli*-ATCC 25922	1 × 10^7^ CFU	CuZn (C23000 & C24700)	86	100%	15 min	26
			63	100%	30 min	
MRSA-NCTC 10442	1 × 10^7^ CFU	CuZn (C24000)	80	4-log	3 h	27
MSSA, VRE, *E. coli*	Not specified	CuZn	60 & 70	90–100%	10 weeks	79
*E. coli*-O157:H7	1 × 10^7^ CFU	CuSi (C65500)	97	100%	65 min	81
		CuAlNi (C61500)	90	100%	180 min	
		CuSn (C51000)	95	100%	105 min	

a UNS: unified numbering system for metals and alloys; %copper: percentage of copper in alloy; ref.: references.

## Critical remarks and future perspectives

In our review of the literature, it is apparent that the specific experimental conditions play a major role in the results obtained and differences between studies make comparison difficult. We have noted differences between studies in the type of microorganism selected including genus, species and strain, and few papers provide justification for their choice. Microbial cultures are often obtained from collections such as the UK's National Collection of Typed Cultures (NCTC) or the American Typed Culture Collection (ATCC) in the US. While the use of these authenticated strains is essential to enable intra-laboratory comparison, there are two important issues to bear in mind: (i) in many cases, the extent of laboratory culture (and hence the extent of genomic evolution as a result of domestication) is undocumented and (ii) the same strains maintained in different laboratories for many years may exhibit genetic differences.^88–90^ This has implications for reproducibility within, and comparison between, studies. It also must be borne in mind that strains within a species are often heterogeneous, and therefore, a single strain is unlikely to be representative of the species as a whole.^91^ Thus, it is advisable to use multiple strains and including freshly isolated (and minimally sub-cultured) clinical isolates as well as standard strains from culture collections.

Studies also differ in the phase of growth microorganisms are harvested for testing, with some using stationary phase cultures where growth is slowed and others using exponential phase cultures where replication rates are maximal. The differences in the physiological state of the microbial population will undoubtedly affect their susceptibility to antibacterials; indeed some studies have documented the differences.^36,78,92^ Other experimental conditions which vary between studies and are known to affect microbial susceptibility to copper include factors that affect inoculum-drying time, like temperature, airflow and humidity.^21,88,93–95^ Issues with the lack of ‘real world’ conditions in the industrial standard tests (*e.g.*, ISO 22196, ISO 21702, ISO 846) and the vast collection of modified protocols that now exist as a result have been previously acknowledged and adequately discussed.^21,93,96,97^ In addition, the need to take into account the effect of cleaning protocols, surface soiling and wear on material efficacy has been documented.^93,97^ We reiterate previous recommendations calling for updated industrial standard tests which apply environmental parameters more reflective of actual conditions within the healthcare setting.

## Conflicts of interest

There are no conflicts to declare.

## Supplementary Material
